# Ultra-Processed Food Intakes Are Associated with Depression in the General Population: The Korea National Health and Nutrition Examination Survey

**DOI:** 10.3390/nu15092169

**Published:** 2023-05-01

**Authors:** Sunghee Lee, Myungjin Choi

**Affiliations:** Department of Food and Nutrition, College of Health Science, Kangwon National University, Samcheok 25949, Republic of Korea; soon6920@naver.com

**Keywords:** ultra-processed food, depression, general population, mental health

## Abstract

Depression is the most common mental illnesses worldwide. The consumption of ultra-processed food (UPF) has increased globally due to its affordability and convenience; however, only a few studies have investigated the link between UPF intake and depression in the general population. We investigated the associations between UPF and depression using the Korea National Health and Nutrition Examination Survey. A total of 9463 individuals (4200 males and 5263 females) aged above 19 years old participated in this study. The prevalence of depression was identified using the Patient Health Questionnaire-9. Dietary intake was assessed through a 24-h recall interview. The percentage of energy from UPFs was ascertained based on the NOVA classification. The associations between the quartile ranges of UPF intake and depression were estimated using logistic regression models. Individuals in the highest quartile had a 1.40 times higher likelihood of having depression, with marginal significance (95% confidence intervals (CIs) = 1.00–1.96). In a sex-specific stratification, only females demonstrated a significant association (odds ratio (OR) = 1.51, 95% CI 1.04–2.21), even after adjusting for confounders (*p*-value for trend = 0.023). Our findings revealed a significant association between higher UPF intake and depression among females but not among males in the Korean general population.

## 1. Introduction

Depression is among the most prevalent mental disorders worldwide [1]. A meta-analysis demonstrated that the burden of depression was 12.9%, particularly higher in females by 14.4% [2]. A general population-based study among adults in the United States (US) showed that the prevalence of depression increased from 8.5% in 2017–2018 to 27.8% in 2020 [3]. Among Korean adults, the prevalence of depression has been increased from 4.3% in 2018 to 5.2% in 2020 [4]. Also, depression increases not only the risk of suicide deaths [1] but also the risk of metabolic syndrome [5].

As a modifiable risk factor, dietary intervention mitigates the risk of depression. The recent advances in food technologies including food packaging, preparation, and extension of shelf-life made possible convenient food packages such as home meal replacements, meal kits, and ready-to-cook meals. Due to its palatability and affordability, the intake of ultra-processed foods (UPFs) intake has increased globally. Particularly, previous studies reported persistent increases in UPFs intake among adults in the US from 53.5% in 2001–2002 to 57.0% in 2017–2018 [6], and among adults in the United Kingdom (UK) from 48.6% in 2009–2010 [7] to 56.8% in 2008–2014 [8]. By contrast, Asian countries reported lower proportions. For example, previous studies have reported the percentage of the UPF intake among Korean adults was 26.8% [9] and 25.1% [10] in 2016 and 2018, respectively. Among Italians, a median UPF intake was approximately 10% [11] due to their characterized Mediterranean diet. Evolving evidence demonstrated that high UPF intake has been associated with mortality [12], obesity [13,14], hypertension [15], diabetes [16], cardiovascular disease [17,18], and dementia [19]. This increased risk of developing chronic diseases was attributed to high sodium intake, excessive sugar intake, high fat intake, and use of food additives [20]. Furthermore, these highly processed foods are less likely to contain fiber, vitamins, and minerals that are commonly found in fresh vegetables and fruits. Therefore, high UPF intake affects the physiology of individuals.

However, existing evidence supporting the association between the psychological aspects of individuals and UPF intake, especially depression, is limited. In addition, only a few studies have examined this link. In France, a previous study among 26,730 individuals aged from 18–86 years old with over 5.4 years of follow-up reported a link between UPF and a higher risk of developing depressive [21]. Particularly, this study demonstrated that the risk of depression was enlarged by 1.21 times as 10% of UPF intake increased (hazard ratio (HR) = 1.21, 95% confidence intervals (CIs) = 1.15–1.27) [21]. Another study in Spain examining 14,907 young adults aged about 36.7 years showed that participants in the highest quartile of UPF intake had a 1.33 times higher likelihood of developing depression than those in the lowest quartile (HR = 1.33, 95% CI 1.07–1.64) [22]. These studies suggested the potential biological links between UPF intake and depression through not only nutritional aspects but also non-nutritious food additives. Hence, more epidemiological evidence needs to be accumulated to elucidate this link.

Thus, we aimed to examine the associations between UPF intake and depression among 9463 participants (4200 males and 5263 females) aged 20 years or older in a general population using the Korea National Health and Nutrition Examination Survey (KNHANES).

## 2. Materials and Methods

### 2.1. Study Population

This study used the data derived from the KNHANES by the Korea Centers for Disease Control and Prevention. The KNHANES was regularly performed to monitor the dietary intakes, health status, and health-related factors in a nationally representative sample. All the participants signed an informed consent. We followed the Declaration of Helsinki guidelines and received approval from the Institutional Review Board (2018-01-03-2C-A, 2018-01-03-P-A). This survey has an exemption by the Bioethics Act of 2016 with the purpose for the public well-being.

**Figure 1:**
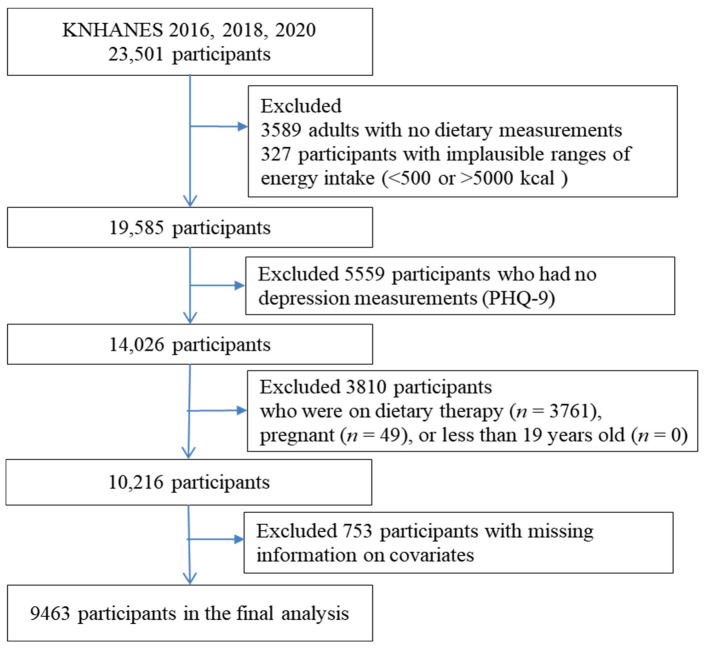
Flow diagram of the study participants.

Of the 23,501 participants included in 2016, 2018, and 2020 KNHANES, 3589 with no data on dietary intake, 327 with implausible daily energy intake (such as <500 or >5000 kcal), and 5559 with no Patient Health Questionnaire-9 (PHQ-9) scores were excluded. Of the remaining 14,026 participants, 3810 who were receiving dietary therapy (*n* = 3761) and were pregnant (*n* = 49) at the time of the study were excluded. In addition, 753 with missing data on body mass index (BMI, *n* = 74), physical activity (PA, *n* = 21), education (*n* = 4), smoking (*n* = 18), hypertension (*n* = 48), diabetes (*n* = 460), and UPF (*n* = 128) were excluded. Hence, 9463 adults were in the study. A flowchart of this process is illustrated in Figure 1.

### 2.2. Definition of Depression

The PHQ-9 was used to identify cases of depression. It comprised nine questions. The scores of PHQ-9 ranged from 0 to 27, with a score of 10 or higher indicating depression [23]. The validity and reliability of the PHQ-9 were previously confirmed [24,25]. The PHQ-9 was administered during the 2016, 2018, and 2020 KNHANES.

### 2.3. Dietary Measurement

To order to determine the dietary intake, professionally trained research staff interviewed those participants and helped them to complete a 24-h recall examination. The nutrient and energy intakes were calculated using the dietary intake data from the Rural Development Administration of Korea database [26]. More than 4000 food items were classified according to the NOVA classification to examine the UPF intake [27,28]. As highlighted by Monteiro and colleagues, the NOVA system has classified food items into four groups: (1) minimally processed or unprocessed foods, (2) culinary ingredients, (3) processed foods, and (4) ultra-processed foods [27,28]. After ascertaining the UPF group, the percentages of total energy intake from the consumption of UPF (%UPF) were calculated.

### 2.4. Covariates

All participants completed the study questionnaires and underwent physical examinations in two large-sized buses as mobile examination centers. Physical activity (PA) was determined using the Global Physical Activity Questionnaire (GPAQ), with moderate or vigorous activities and total activity [29]. Based on the time spent performing these activities and the intensity of moderate or vigorous activities, the participants were asked about activities at work, for recreation, and when traveling to and from certain places, and the frequency and duration of walking during a usual week [29,30]. The validity and reliability of the Korean version of the GPAQ have been verified [30]. According to the PA guidelines [31], adults should perform more than 150 min of moderate–intensity, 75 min of vigorous–intensity, or the corresponding analogous combined time [30]. Smoking status was classified as nonsmoker, ex-smoker, or current smoker. “Alcohol drinker” was defined as an individual who consumed alcohol more than once per month during the past year; “alcohol non-drinker” was defined as an individual who consumed alcohol less than once per month. Individuals with “hypertension” were identified as those with a systolic blood pressure of ≥140 mmHg, with a diastolic blood pressure of ≥90 mmHg, or using antihypertensive medications. Participants with “diabetes mellitus” were defined as those with a fasting glucose level of ≥126 mg/dL, who were diagnosed by a medical doctor or were using antidiabetic medications.

### 2.5. Statistical Analysis

To investigate the KNHANES data derived using the complex sampling method, weighted survey analyses such as surveymeans, surveyfreq, and surveyreg, were used to examine the data. To evaluate the different characteristics according to the quartiles of UPF, survey regression models were used with a *p*-value for trend. Additionally, to assess the associations between UPF intake and depression, multivariable logistic regression models (e.g., surveylogistic) were used after adjusting for confounding variables including age, BMI, education, PA, alcohol drinking, smoking, hypertension, and diabetes. All analyses were performed using SAS version 9.4, with the significant level as *p*-value < 0.05.

## 3. Results

Table 1 demonstrates the general characteristics of the 9463 participants and the comparisons of sex-specific characteristics among 4200 males and 5263 females between those with and without depression. Among males, smoking status (*p* < 0.001) and diabetes (*p* = 0.003) were significantly associated with depression; among males with depression, 53.79% were current smokers, and a significantly higher proportion had diabetes. On the other hand, among females, BMI (*p* = 0.008), education (*p* = 0.003), smoking (*p* < 0.001), diabetes (*p* < 0.001), and UPF intake (*p* = 0.002) were significantly associated with depression.

Table 2 lists the general characteristics of 4200 males according to the quartile ranges of UPF intake. Among the 133 males with depression, those in the higher quartile groups were younger (*p* < 0.001). In contrast, among the 4067 males without depression, those in the higher quartile groups were significantly younger (*p* < 0.001), had a higher BMI (*p* = 0.004), had higher education level (*p* < 0.001), were current smokers (*p* < 0.001), were alcohol drinkers (*p* < 0.001), and were less likely to have diabetes (*p* = 0.012) and hypertension (*p* < 0.001).

Table 3 shows the characteristics of 5263 females according to the quartile of UPF intake. Among the 312 females with depression, those in the higher quartile groups were significantly younger (*p* < 0.001), had higher education level (*p* < 0.001), were current drinkers (*p* = 0.007), and were less likely to have hypertension (*p* = 0.001) and diabetes (*p* = 0.023). Among 4951 females without depression, those in the higher quartile groups were significantly younger (*p* < 0.001), had a lower BMI (*p* < 0.001), had higher education level (*p* < 0.001), were more physically active (*p* = 0.026), were current smokers (*p* < 0.001), were current drinkers (*p* < 0.001), and were less likely to have diabetes (*p* < 0.001) and hypertension (*p* < 0.001).

Table 4 presents the nutrient intake of 4200 males and 5263 females according to the quartile of UPF intake. In both males and females, the intakes of sugar, fat, saturated fat, and dietary sodium were positively increased as the consumption of UPFs increased (*p* < 0.001, respectively), except for the carbohydrate and protein intakes. By contrast, the intakes of vegetables and fruits were significantly decreased as the UPF intake increased (*p* < 0.001, respectively). The nutrient intake according to individuals with and without depression within 4200 males and 5263 females were examined and were shown in consistent results (Supplemental Tables S1 and S2). 

Table 5 demonstrates the association between UPF intake and depression after considering the confounding factors. In the total population, individuals in the highest quartile were 1.40 times more likely to have depression, with marginally significance (95% CI = 1.00–1.96). Males showed no association, whereas females demonstrated a significant association, indicating that females in the highest quartile of UPF were 1.51 times more likely to have depression (95% CI 1.04, 2.21), even after adjusting for age, BMI, education, PA, alcohol drinking, smoking, diabetes, and hypertension (*p* for trend = 0.023).

## 4. Discussion

In this cross-sectional study, we found that the burden of depression in the Korean population from 2016 to 2020 was 4.40%, indicating that it was more prevalent in females than males (5.90% vs. 3.04%). Moreover, we identified that the average percentage of energy from UPFs in the Korean general population was approximately 27.49%. Furthermore, participants with higher UPF intake had higher sugar, fat, saturated fat, and dietary sodium intake (*p* for trend < 0.001, respectively) but lower intakes of vegetables and fruits (*p* for trend < 0.001, respectively). Furthermore, females in the highest quartile of UPF intake had a 1.51 times higher likelihood of having depression, after adjusting for confounders (OR = 1.51, 95% CI 1.04–2.21, *p* for trend = 0.023).

Our study was the first to examine the associations between UPF intake and depression in a general population from an Asian country. The percentage of energy intake from the consumption of UPFs varies among different cultural backgrounds; the US reported an energy intake of 57.0% in 2017–2018 [6], the UK indicated 56.8% in 2008–2014 [8], and Italy had approximately 10% due to the Mediterranean diet [11].

Few studies that previously assessed the association between UPF intake and depression reported results that were concurrent with our findings [21,22,32,33]. A previous study of 26,730 participants with a follow-up of 5.4 years reported the association of increased risk of depressive symptoms with UPF intake, showing that a 10% increase in UPF intake was associated with 1.21 times higher risk of developing depression (95% CI = 1.15–1.27) [21]. Another study among 14,907 adults demonstrated an increased risk of depression in the highest quartile of UPF compared with that in the lowest quartile of UPF (HR = 1.33, 95% CI 1.07–1.64) [22]. A recent study among Italian participants demonstrated a significant association between UPF intake and depressive symptoms (OR=2.04, 95% CI 1.04–4.01) [33]. Furthermore, a meta-analysis indicated the link between UPF intake and higher risk of depression (RR = 1.28, 95% CI = 1.19–1.38) [32]. However, different from the two studies, their results adopted various methods in defining UPFs, including not only the NOVA classification of food items but also fast foods, Western dietary patterns, and sweetened foods.

Our study participants showed a relatively lower prevalence of depression. To ascertain those with depression among the study participants, we used the PHQ-9, which has been confirmed to be valid and reliable [24,25]. A study using the NHANES data with PHQ-9 among 34,963 participants aged 18 years or older showed that the prevalence of depression was 8.1% (males: 6.5% and females: 9.6%) between 2015 and 2016 [23]. Based on the responses to the PHQ-9, this current study demonstrated that the prevalence of depression was 4.40% (males: 3.04% and females: 5.90%) between 2016 and 2020. Furthermore, females consistently demonstrated a higher prevalence of depression, which may explain in part the differences in the significance of depression associated with high UPF intake.

We also observed sex-specifically differences in the profiles of risk factors for depression and their associations with UPF intake. The general characteristics among males with or without depression were not different except for smoking status and prevalence of diabetes. However, females with and without depression tended to show significant differences in BMI, education level, smoking status, the prevalence of diabetes, and UPF intake. Sex differences in depression were consistent with a previous study [34].

More importantly, in addition to the significantly higher intake of UPFs among those with depression that we observed above, our findings revealed that according to the quartiles of UPF intake, both males and females demonstrated significantly increasing trends in nutrient intakes in total energy, sugar, fat, and saturated fat, dietary sodium, but significantly decreasing trends in food groups of vegetables and fruits. However, no significant trends were shown in carbohydrate and protein intakes.

The mechanisms underlying the psychological aspects linked to UPFs have not yet been elucidated. However, previous studies have attempted to explain this association. First, high UPF intakes are deficient in bioactive micronutrients such as minerals and vitamins due to limited whole food constituents from vegetables or fruits. This deficiency may subsequently be attributable to the increased risk of depression [21]. Second, high UPF intakes aggravate the disturbance of gut microbiota balance, leading to gut dysbiosis, followed by detrimental effects on the gut–brain axis, which reduce the production of neurotransmitters such as serotonin [32,35]. Moreover, high UPF intake causes hypothalamic-pituitary adrenal (HPA) dysregulation, which affects appetite hormones and regulatory neurotransmitter signals [13,22]. Third, gut dysbiosis from high UPF intake stimulates pro-inflammatory cytokines [36], leading to increased concentrations of high-sensitivity C-reactive protein [37]. Finally, food additives for flavoring, coloring, palatable enhancers, and emulsifiers [38], or byproducts and contaminants in the production of UPFs may induce detrimental effects by disrupting endocrine signals or homeostatic regulatory pathways [39,40]. Thus, further studies on these links are warranted.

This study has several strengths and limitations. Firstly, the study sample was relatively large with 9463 participants; therefore, it had enough statistical power to detect the differences even in the sex-specific stratified groups. Secondly, the data were obtained from the general population through a nationally representative survey, which enables the generalizability of the study results to other populations. However, this study also has limitations. It used a cross-sectional design which limited to establish a causal relationship. Moreover, data on dietary intake were obtained through a single 24-h dietary recall interview, which might have day-to-day variations. However, the day-to-day variations in dietary intake could be mitigated with the person-to-person variations due to the large sample size used in the study.

## 5. Conclusions

In conclusion, our findings revealed a significant association between high UPF intake and depression among 5263 females but not in 4200 males in the general Asian population, even after adjusting for age, BMI, alcohol drinking, smoking, education, hypertension, diabetes, and PA. Based on these findings, further studies are warranted.

## Figures and Tables

**Table 1:** General characteristics of the study participants according to depression (*n* = 9463).

	Total (*n* = 9463)		*p*	Male (*n* = 4200)		*p*	Female (*n* = 5263)		*p*
	Depression	No Depression		Depression	No Depression		Depression	No Depression	
	*n* = 445 (4.40%)	*n* = 9018 (95.60%)		*n* = 133 (3.04%)	*n* = 4067 (96.96%)		*n* = 312 (5.90%)	*n* = 4951 (94.10%)	
Age, years	45.81 ± 1.07	46.97 ± 0.29	0.276	44.06 ± 1.61	46.17 ± 0.36	0.195	46.80 ± 1.35	47.87 ± 0.33	0.433
BMI, kg/m^2^	24.01 ± 0.24	23.76 ± 0.05	0.324	24.58 ± 0.47	24.47 ± 0.07	0.825	23.68 ± 0.27	22.96 ± 0.07	0.008
Education, %									
<High school	31.57	21.12	<0.001	23.72	16.54	0.094	36.01	26.30	0.003
High school	36.08	36.95		39.82	39.19		33.96	34.42	
>High school	32.35	41.93		36.46	44.27		30.03	39.28	
Physical activity, %									
Active	37.02	44.04	0.021	39.79	46.88	0.171	35.45	40.83	0.125
Inactive	62.98	55.96		60.21	53.12		64.55	59.17	
Smoking status, %									
Current smokers	32.75	21.31	<0.001	53.79	35.33	0.001	20.82	5.43	<0.001
Ex-smokers	19.21	22.48		30.35	37.19		12.90	5.84	
Non-smokers	48.05	56.21		15.87	27.48		66.28	88.73	
Alcohol drinker, %	55.92	59.11	0.298	74.01	71.32	0.557	45.68	45.29	0.919
Hypertension, %	27.05	26.10	0.696	31.40	29.15	0.623	24.59	22.66	0.463
Diabetes mellitus, %	14.80	8.22	<0.001	18.12	9.61	0.003	12.92	6.64	<0.001
UPF, %	31.11 ± 1.28	27.32 ± 0.29	0.003	33.29 ± 2.41	29.07 ± 0.41	0.080	29.87 ± 1.41	25.34 ± 0.35	0.002
UPF energy, kcal	615.01 ± 35.52	590.64 ± 8.36	0.504	739.06 ± 68.58	713.87 ± 12.63	0.717	544.72 ± 40.21	451.16 ± 7.87	0.023
Total energy, kcal/day	1875.81 ± 48.46	2037.47 ± 12.21	0.001	2209.56 ± 76.30	2326.86 ± 17.07	0.131	1686.71 ± 56.51	1709.92 ± 11.87	0.686

Mean ± SE; BMI, body mass index.

**Table 2:** Means and frequencies of the general characteristics of the males according to ultra-processed food.

	Males (*n* = 4200)									
	Depression (*n* = 133)					No Depression (*n* = 4067)				
	Q1	Q2	Q3	Q4	*p*	Q1	Q2	Q3	Q4	*p*
	*n* = 28 (13.32%)	*n* = 27 (23.94%)	*n* = 32 (25.30%)	*n* = 46 (37.44%)		*n* = 891 (18.55%)	*n* = 943 (22.81%)	*n* = 1069 (26.30%)	*n* = 1164 (32.34%)	
Age, years	52.52 ± 5.53	39.30 ± 2.80	42.66 ± 3.28	45.03 ± 2.18	<0.001	53.94 ± 0.71	48.54 ± 0.64	45.25 ± 0.57	40.81 ± 0.49	<0.001
BMI, kg/m^2^	22.65 ± 0.78	25.82 ± 0.83	25.05 ± 0.99	24.15 ± 0.66	0.891	24.14 ± 0.14	24.28 ± 0.13	24.67 ± 0.13	24.65 ± 0.14	0.004
Education										
<High school	35.87	15.91	14.75	30.46	0.581	28.42	18.85	12.58	11.32	<0.001
High school	39.58	38.30	46.70	36.24		32.57	35.86	39.86	44.78	
>High school	24.55	45.78	38.56	33.30		39.01	45.29	47.55	43.90	
Physical activity, %										
Active	19.09	45.61	39.36	43.74	0.370	45.71	47.38	46.62	47.42	0.915
Inactive	80.91	54.39	60.64	56.26		54.29	52.62	53.38	52.58	
Smoking status, %										
Current smokers	45.19	36.08	52.58	68.98	0.306	26.63	28.36	37.58	43.40	<0.001
Ex-smokers	35.13	43.87	29.20	20.77		44.23	41.72	35.85	31.04	
Non-smokers	19.68	20.06	18.22	10.25		29.14	29.92	26.57	25.56	
Alcohol drinker, %	71.39	74.72	67.71	78.73	0.789	64.00	67.70	72.39	77.19	<0.001
Hypertension, %	41.35	31.30	20.22	35.46	0.452	36.88	27.81	27.03	27.38	<0.001
Diabetes mellitus, %	13.56	11.04	20.34	22.77	0.565	12.76	9.80	9.15	8.05	0.012

Mean ± SE; BMI, body mass index.

**Table 3:** Means and frequencies of the general characteristics of the females according to ultra-processed food.

	Females (*n* = 5263)									
	Depression (*n* = 312)					No Depression (*n* = 4951)				
	Q1	Q2	Q3	Q4	*p*	Q1	Q2	Q3	Q4	*p*
	*n* = 73 (21.60%)	*n* = 72 (21.13%)	*n* = 76 (23.84%)	*n* = 91 (33.44%)		*n* = 1357 (24.39%)	*n* = 1317 (25.27%)	*n* = 1193 (25.41%)	*n* = 1084 (24.93%)	
Age, years	60.39 ± 2.66	48.28 ± 2.33	46.87 ± 2.40	37.03 ± 1.75	<0.001	56.94 ± 0.51	50.11 ± 0.55	44.82 ± 0.51	39.84 ± 0.57	<0.001
BMI, kg/m^2^	24.55 ± 0.46	22.87 ± 0.71	23.74 ± 0.57	23.60 ± 0.47	0.445	23.51 ± 0.11	23.01 ± 0.12	22.77 ± 0.13	22.57 ± 0.14	<0.001
Education										
<High school	66.91	33.81	33.43	19.29	<0.001	46.64	27.63	17.53	13.97	<0.001
High school	17.65	41.09	26.79	45.10		30.05	34.82	34.59	38.12	
>High school	15.44	25.10	39.78	35.61		23.31	37.55	47.88	47.90	
Physical activity, %										
Active	41.47	42.15	31.06	30.45	0.392	38.05	40.20	44.91	40.04	0.026
Inactive	58.53	57.85	68.94	69.55		61.95	59.81	55.09	59.96	
Smoking status, %										
Current smokers	15.15	12.11	22.09	29.09	0.103	3.81	4.69	4.06	9.18	<0.001
Ex-smokers	6.05	15.60	16.20	13.27		3.43	6.09	5.94	7.82	
Non-smokers	78.80	72.29	61.71	57.64		92.76	89.22	90.00	83.00	
Alcohol drinker, %	26.60	47.49	43.84	58.17	0.007	30.90	43.81	48.46	57.66	<0.001
Hypertension, %	41.61	29.34	22.64	11.99	0.001	35.59	24.68	17.22	13.50	<0.001
Diabetes mellitus, %	20.68	15.81	14.74	4.78	0.023	12.06	5.68	4.87	4.11	<0.001

Mean ± SE; BMI, body mass index.

**Table 4:** Means of nutrient intakes of the study participants according to the quartile range of ultra-processed food intakes (*n* = 9463).

	Males (*n* = 4200)					Females (*n* = 5263)				
	Q1	Q2	Q3	Q4	*p*	Q1	Q2	Q3	Q4	*p*
	*n* = 919 (18.39%)	*n* = 970 (22.84%)	*n* = 1101 (26.27%)	*n* = 1210 (32.50%)		*n* = 1430 (24.22%)	*n* = 1389 (25.03%)	*n* = 1269 (25.32%)	*n* = 1175 (25.43%)	
UPF energy, kcal	93.27 ± 3.08	333.32 ± 5.91	698.12 ± 8.99	1347.65 ± 19.51	<0.001	64.04 ± 1.74	251.52 ± 3.58	511.46 ± 6.86	978.01 ± 16.87	<0.001
Total energy, kcal/day	2001.36 ± 27.92	2193.04 ± 32.99	2398.40 ± 28.44	2536.30 ± 29.36	<0.001	1547.14 ± 18.17	1685.45 ± 20.61	1762.20 ± 21.22	1831.62 ± 25.94	<0.001
Carbohydrate, g	322.26 ± 4.59	328.48 ± 4.58	338.22 ± 4.14	324.77 ± 4.07	0.639	263.91 ± 3.29	267.1 ± 3.46	262.62 ± 3.14	264.62 ± 3.50	0.868
Sugar, g	49.59 ± 1.33	62.16 ± 1.57	69.19 ± 1.55	70.22 ± 1.46	<0.001	47.35 ± 1.13	57.53 ± 1.36	61.55 ± 1.23	64.45 ± 1.37	<0.001
Protein, g	79.67 ± 1.55	85.76 ± 1.64	89.20 ± 1.44	82.75 ± 1.23	0.213	56.74 ± 0.96	63.15 ± 1.03	64.96 ± 0.99	59.02 ± 1.02	0.056
Fat, g	41.73 ± 1.41	53.24 ± 1.77	59.09 ± 1.29	58.04 ± 1.12	<0.001	29.04 ± 0.75	38.84 ± 0.93	45.44 ± 0.94	46.91 ± 1.12	<0.001
Saturated fat, g	12.29 ± 0.48	16.55 ± 0.59	18.83 ± 0.43	20.03 ± 0.43	<0.001	8.37 ± 0.24	12.05 ± 0.33	14.70 ± 0.32	17.06 ± 0.47	<0.001
Dietary sodium, mg	3712.80 ± 80.11	3863.48 ± 76.64	4214.23 ± 70.02	4271.37 ± 72.22	<0.001	2557.88 ± 49.32	2852.45 ± 55.58	3053.09 ± 61.80	3182.66 ± 61.09	<0.001
Food Groups										
Vegetables, g	394.40 ± 8.49	353.28 ± 7.77	342.59 ± 7.16	275.66 ± 5.99	<0.001	309.21 ± 6.18	282.97 ± 5.34	253.56 ± 5.50	203.41 ± 5.59	<0.001
Fruits, g	286.27 ± 14.47	254.31 ± 12.29	224.84 ± 11.68	164.82 ± 9.74	<0.001	292.48 ± 10.51	268.06 ± 13.27	223.78 ± 10.00	179.66 ± 8.20	<0.001

Mean ± SE; BMI, body mass index.

**Table 5:** Odds ratios associated with the quartile ranges of the ultra-processed foods on depression (*n* = 9463).

	Odds Ratio (95% Confidence Intervals)				*p*-Value for Trend
	Quartile Range				
	Q1	Q2	Q3	Q4	
Total					
*n* (%)	2349 (21.17%)	2359 (23.89%)	2370 (25.81%)	2385 (29.13%)	
UPF% range	[0, 9.17]	[9.17, 21.22]	[21.22, 37.76]	[35.76, 100]	
OR (95% CI)	1.00 (Ref)	1.18 (0.83, 1.66)	1.21 (0.84, 1.75)	1.40 (1.00, 1.96)	0.066
Males					
*n* (%)	919 (45.47%)	970 (50.06%)	1101 (53.27%)	1210 (58.40%)	
UPF% range	[0, 9.18]	[9.18, 21.22]	[21.22, 37.75]	[35.75, 100]	
OR (95% CI)	1.00 (Ref)	1.47 (0.74, 2.91)	1.23 (0.64, 2.39)	1.34 (0.70, 2.55)	0.624
Females					
*n* (%)	1430 (54.53%)	1389 (49.94%)	1269 (46.73%)	1175 (41.60%)	
UPF% range	[0, 9.17]	[9.17, 21.21]	[21.21, 37.76]	[35.76, 100]	
OR (95% CI)	1.00 (Ref)	1.04 (0.70, 1.54)	1.24 (0.81, 1.91)	1.51 (1.04, 2.21)	0.023

Adjusted for age, body mass index, education, physical activity, smoking, alcohol drinking, hypertension, and diabetes mellitus.

## Data Availability

The data presented in this study are openly available at http://knhanes.kdca.go.kr.

## Supplementary Materials

The following supporting information can be downloaded at: https://www.mdpi.com/article/10.3390/nu15092169/s1; Table S1: Means of Nutrients of the Males according to Ultra-processed Food Intakes; Table S2: Means of Nutrients of the Females according to Ultra-processed Food Intakes.

## References

[1] WHO Depression. https://www.who.int/news-room/fact-sheets/detail/depression.

[2] Lim G.Y., Tam W.W., Lu Y., Ho C.S., Zhang M.W., Ho R.C. (2018). Prevalence of Depression in the Community from 30 Countries between 1994 and 2014. Sci. Rep..

[3] Ettman C.K., Abdalla S.M., Cohen G.H., Sampson L., Vivier P.M., Galea S. (2020). Prevalence of Depression Symptoms in US Adults Before and During the COVID-19 Pandemic. JAMA Netw. Open.

[4] Lee E.J., Kim S.J. (2023). Prevalence and Related Factors of Depression Before and During the COVID-19 Pandemic: Findings From the Korea National Health and Nutrition Examination Survey. J. Korean Med. Sci..

[5] Ferriani L.O., Silva D.A., Molina M., Mill J.G., Brunoni A.R., da Fonseca M.J.M., Moreno A.B., Bensenor I.M., de Aguiar O.B., Barreto S.M. (2022). Depression is a risk factor for metabolic syndrome: Results from the ELSA-Brasil cohort study. J. Psychiatr. Res..

[6] Juul F., Parekh N., Martinez-Steele E., Monteiro C.A., Chang V.W. (2022). Ultra-processed food consumption among US adults from 2001 to 2018. Am. J. Clin. Nutr..

[7] Rauber F., Chang K., Vamos E.P., da Costa Louzada M.L., Monteiro C.A., Millett C., Levy R.B. (2021). Ultra-processed food consumption and risk of obesity: A prospective cohort study of UK Biobank. Eur. J. Nutr..

[8] Rauber F., da Costa Louzada M.L., Steele E.M., Millett C., Monteiro C.A., Levy R.B. (2018). Ultra-Processed Food Consumption and Chronic Non-Communicable Diseases-Related Dietary Nutrient Profile in the UK (2008–2014). Nutrients.

[9] Sung H., Park J.M., Oh S.U., Ha K., Joung H. (2021). Consumption of Ultra-Processed Foods Increases the Likelihood of Having Obesity in Korean Women. Nutrients.

[10] Shim J.S., Shim S.Y., Cha H.J., Kim J., Kim H.C. (2022). Association between Ultra-processed Food Consumption and Dietary Intake and Diet Quality in Korean Adults. J. Acad. Nutr. Diet..

[11] Bonaccio M., Di Castelnuovo A., Costanzo S., De Curtis A., Persichillo M., Sofi F., Cerletti C., Donati M.B., de Gaetano G., Iacoviello L. (2021). Ultra-processed food consumption is associated with increased risk of all-cause and cardiovascular mortality in the Moli-sani Study. Am. J. Clin. Nutr..

[12] Schnabel L., Kesse-Guyot E., Alles B., Touvier M., Srour B., Hercberg S., Buscail C., Julia C. (2019). Association Between Ultraprocessed Food Consumption and Risk of Mortality Among Middle-aged Adults in France. JAMA Intern. Med..

[13] Lane M.M., Davis J.A., Beattie S., Gomez-Donoso C., Loughman A., O’Neil A., Jacka F., Berk M., Page R., Marx W. (2021). Ultraprocessed food and chronic noncommunicable diseases: A systematic review and meta-analysis of 43 observational studies. Obes. Rev..

[14] Beslay M., Srour B., Mejean C., Alles B., Fiolet T., Debras C., Chazelas E., Deschasaux M., Wendeu-Foyet M.G., Hercberg S. (2020). Ultra-processed food intake in association with BMI change and risk of overweight and obesity: A prospective analysis of the French NutriNet-Sante cohort. PLoS Med..

[15] Shim S.Y., Kim H.C., Shim J.S. (2022). Consumption of Ultra-Processed Food and Blood Pressure in Korean Adults. Korean Circ. J..

[16] Srour B., Fezeu L.K., Kesse-Guyot E., Alles B., Debras C., Druesne-Pecollo N., Chazelas E., Deschasaux M., Hercberg S., Galan P. (2020). Ultraprocessed Food Consumption and Risk of Type 2 Diabetes Among Participants of the NutriNet-Sante Prospective Cohort. JAMA Intern. Med..

[17] Zhang Z., Jackson S.L., Martinez E., Gillespie C., Yang Q. (2021). Association between ultraprocessed food intake and cardiovascular health in US adults: A cross-sectional analysis of the NHANES 2011—2016. Am. J. Clin. Nutr..

[18] Chen X., Zhang Z., Yang H., Qiu P., Wang H., Wang F., Zhao Q., Fang J., Nie J. (2020). Consumption of ultra-processed foods and health outcomes: A systematic review of epidemiological studies. Nutr. J..

[19] Li H., Li S., Yang H., Zhang Y., Zhang S., Ma Y., Hou Y., Zhang X., Niu K., Borne Y. (2022). Association of Ultraprocessed Food Consumption With Risk of Dementia: A Prospective Cohort Study. Neurology.

[20] Lustig R.H. (2020). Ultraprocessed Food: Addictive, Toxic, and Ready for Regulation. Nutrients.

[21] Adjibade M., Julia C., Alles B., Touvier M., Lemogne C., Srour B., Hercberg S., Galan P., Assmann K.E., Kesse-Guyot E. (2019). Prospective association between ultra-processed food consumption and incident depressive symptoms in the French NutriNet-Sante cohort. BMC Med..

[22] Gomez-Donoso C., Sanchez-Villegas A., Martinez-Gonzalez M.A., Gea A., Mendonca R.D., Lahortiga-Ramos F., Bes-Rastrollo M. (2020). Ultra-processed food consumption and the incidence of depression in a Mediterranean cohort: The SUN Project. Eur. J. Nutr..

[23] Kauffman K., Horvat Davey C., Dolata J., Figueroa M., Gunzler D., Huml A., Pencak J., Sajatovic M., Sehgal A.R. (2021). Changes in Self-Reported Depressive Symptoms Among Adults in the United States From 2005 to 2016. J. Am. Psychiatr. Nurses Assoc..

[24] Han C., Jo S.A., Kwak J.H., Pae C.U., Steffens D., Jo I., Park M.H. (2008). Validation of the Patient Health Questionnaire-9 Korean version in the elderly population: The Ansan Geriatric study. Compr. Psychiatry.

[25] Park S.J., Choi H.R., Choi J.H., Kim K.W., Hong J.P. (2010). Reliability and Validity of the Korean Version of the Patient Health Questionnaire-9 (PHQ-9). Anxiety Mood.

[26] Park S.-H., Kim S.-N., Lee S., Choe J.-S., Choi Y. (2018). Development of 9 th Revision Korean Food Composition Table and Its Major Changes. Korean J. Community Nutr..

[27] Monteiro C.A., Cannon G., Levy R.B., Moubarac J.C., Louzada M.L., Rauber F., Khandpur N., Cediel G., Neri D., Martinez-Steele E. (2019). Ultra-processed foods: What they are and how to identify them. Public Health Nutr..

[28] Monteiro C.A., Cannon G., Moubarac J.C., Levy R.B., Louzada M.L.C., Jaime P.C. (2018). The UN Decade of Nutrition, the NOVA food classification and the trouble with ultra-processing. Public Health Nutr..

[29] WHO Global Physical Activity Questionnaire (GPAQ). https://www.who.int/teams/noncommunicable-diseases/surveillance/systems-tools/physical-activity-surveillance.

[30] Lee J., Lee C., Min J., Kang D.W., Kim J.Y., Yang H.I., Park J., Lee M.K., Lee M.Y., Park I. (2020). Development of the Korean Global Physical Activity Questionnaire: Reliability and validity study. Glob. Health Promot..

[31] Piercy K.L., Troiano R.P., Ballard R.M., Carlson S.A., Fulton J.E., Galuska D.A., George S.M., Olson R.D. (2018). The Physical Activity Guidelines for Americans. JAMA.

[32] Mazloomi S.N., Talebi S., Mehrabani S., Bagheri R., Ghavami A., Zarpoosh M., Mohammadi H., Wong A., Nordvall M., Kermani M.A.H. (2022). The association of ultra-processed food consumption with adult mental health disorders: A systematic review and dose-response meta-analysis of 260,385 participants. Nutr. Neurosci..

[33] Godos J., Bonaccio M., Al-Qahtani W.H., Marx W., Lane M.M., Leggio G.M., Grosso G. (2023). Ultra-Processed Food Consumption and Depressive Symptoms in a Mediterranean Cohort. Nutrients.

[34] Kuehner C. (2017). Why is depression more common among women than among men?. Lancet Psychiatry.

[35] Ortega M.A., Alvarez-Mon M.A., Garcia-Montero C., Fraile-Martinez O., Guijarro L.G., Lahera G., Monserrat J., Valls P., Mora F., Rodriguez-Jimenez R. (2022). Gut Microbiota Metabolites in Major Depressive Disorder-Deep Insights into Their Pathophysiological Role and Potential Translational Applications. Metabolites.

[36] Martinez Leo E.E., Segura Campos M.R. (2020). Effect of ultra-processed diet on gut microbiota and thus its role in neurodegenerative diseases. Nutrition.

[37] Lane M.M., Lotfaliany M., Forbes M., Loughman A., Rocks T., O’Neil A., Machado P., Jacka F.N., Hodge A., Marx W. (2022). Higher Ultra-Processed Food Consumption Is Associated with Greater High-Sensitivity C-Reactive Protein Concentration in Adults: Cross-Sectional Results from the Melbourne Collaborative Cohort Study. Nutrients.

[38] Kliemann N., Al Nahas A., Vamos E.P., Touvier M., Kesse-Guyot E., Gunter M.J., Millett C., Huybrechts I. (2022). Ultra-processed foods and cancer risk: From global food systems to individual exposures and mechanisms. Br. J. Cancer.

[39] Montera V., Martins A.P.B., Borges C.A., Canella D.S. (2021). Distribution and patterns of use of food additives in foods and beverages available in Brazilian supermarkets. Food Funct..

[40] Buckley J.P., Kim H., Wong E., Rebholz C.M. (2019). Ultra-processed food consumption and exposure to phthalates and bisphenols in the US National Health and Nutrition Examination Survey, 2013–2014. Environ. Int..

