# Supplementary material for: Ultra-Processed Food Intakes Are Associated with Depression in the General Population: The Korea National Health and Nutrition Examination Survey
Source: Nutrients. 2023 May 1;15(9):2169. doi: 10.3390/nu15092169 (PMC10180546; doi:10.3390/nu15092169)
Supplement: Supplementary file 1 [file nutrients-15-02169-s001.zip › nutrients-2336480-supplementary.pdf]

Supplemental Table S1. Means of Nutrients of the Males according to Ultra-processed Food Intakes.

	Male (n=4,200)									
	Depression (n=133)					No depression (n=4,067)				
	Q1	Q2	Q3	Q4	*p*	Q1	Q2	Q3	Q4	*p*
	n=28 (13.32%)	n=27 (23.94%)	n=32 (25.30%)	n=46 (37.44%)		n=891 (18.55%)	n=943 (22.81%)	n=1,069 (26.30%)	n=1,164 (32.34 %)	
UPF energy, kcal	121.78±28.97	328.52±38.95	626.28±39.85	1297.4±123.37	<0.001	92.63±3.05	333.48±5.96	700.29±9.14	1349.48±19.76	<0.001
Total energy, kcal/day	2324.86±212.16	2236.45±180.04	2195.38±125.61	2160.93±123.76	0.526	1994.07±27.57	2191.61±32.67	2404.52±28.82	2549.94±29.81	<0.001
Carbohydrate, g	339.71±15.50	352.66±36.95	308.04±15.83	275.57±17.16	0.007	321.86±4.67	327.69±4.41	339.13±4.22	326.56±4.18	0.366
Sugar, g	48.68±5.53	69.92±11.74	54.87±7.34	47.97±4.85	0.212	49.61±1.36	61.9±1.53	69.62±1.56	71.02±1.49	<0.001
Protein, g	87.78±9.37	89.18±7.62	80.58±7.03	62.69±4.56	<0.001	79.49±1.57	85.65±1.67	89.46±1.47	83.48±1.24	0.0761
Fat, g	65.42±16.95	50.48±6.55	54.11±6.28	42.5±3.54	0.140	41.2±1.37	53.33±1.81	59.24±1.29	58.61±1.15	<0.001
Saturated fat, g	21.29±5.65	14.41±1.73	17.62±2.44	15.33±1.43	0.453	12.09±0.48	16.62±0.6	18.87±0.43	20.2±0.43	<0.001
Dietary sodium, mg	4064.16±487.84	3586.20±428.98	3722.27±429.98	3869.01±319.36	0.980	3704.88±81.09	3872.61±77.63	4229.08±69.80	4285.98±73.34	<0.001
Food Groups										
Vegetables, g	364.59±39.84	324.27±50.79	302.09±43.08	232.78±22.12	0.004	395.08±8.71	354.24±7.86	343.78±7.19	277.13±6.13	<0.001
Fruits, g	132.85±37.28	364.43±61.98	161.62±52.11	80.29±25.93	0.033	290.38±14.79	251.57±12.29	226.55±11.96	167.54±9.97	<0.001

mean±SE; BMI, body mass index

Supplemental Table S2. Means of Nutrients of the Females according to Ultra-processed Food Intakes.

	Female (n=5,263)									
	Depression (n=312)					No depression (n=4,951)				
	Q1	Q2	Q3	Q4	p	Q1	Q2	Q3	Q4	p
	n=73 (21.60%)	n=72 (21.13%)	n=76 (23.84%)	n=91 (33.44%)		n=1,357 (24.39%)	n=1,317 (25.27%)	n=1,193 (25.41%)	n=1,084 (24.93%)	
UPF energy, kcal	52.20±6.90	250.22±17.64	490.56±29.67	1087.57±75.72	<0.001	64.70±1.79	251.58±3.67	512.69±7.08	968.80±16.91	<0.001
Total energy, kcal/day	1416.19±73.99	1638.29±86.36	1649.22±93.99	1918.79±107.12	<0.001	1554.41±18.59	1687.93±21.20	1768.84±21.80	1824.29±26.35	<0.001
Carbohydrate, g	261.39±18.01	262.87±12.74	254.46±13.99	269.49±14.31	0.763	264.05±3.35	267.32±3.60	263.10±3.20	264.21±3.54	0.796
Sugar, g	45.93±5.45	56.17±5.90	52.53±4.11	72.82±6.57	0.003	47.43±1.16	57.60±1.41	62.09±1.28	63.75±1.34	<0.001
Protein, g	46.97±2.05	62.02±4.87	55.73±4.40	58.74±3.51	0.070	57.29±1.00	63.21±1.06	65.50±1.02	59.04±1.06	0.100
Fat, g	19.75±1.63	35.64±3.43	40.27±3.67	50.59±4.11	<0.001	29.55±0.79	39.01±0.95	45.74±0.97	46.61±1.15	<0.001
Saturated fat, g	5.98±0.65	10.49±1.12	13.68±1.20	18.42±1.64	<0.001	8.50±0.26	12.13±0.34	14.76±0.33	16.94±0.48	<0.001
Dietary sodium, mg	2369.31±145.97	2713.06±224.30	2876.60±219.11	2983.51±201.36	0.022	2568.35±50.91	2859.76±57.69	3063.47±63.05	3199.4±64.24	<0.001
Food Groups										
Vegetables, g	318.83±30.19	238.82±27.97	199.15±16.31	201.94±22.05	0.002	308.67±6.26	285.24±5.53	256.78±5.68	203.54±5.74	<0.001
Fruits, g	283.40±49.16	301.14±61.59	160.07±24.65	149.21±23.11	0.004	292.95±10.83	266.52±13.62	226.93±10.42	181.77±8.60	<0.001

mean±SE; BMI, body mass index
